# Effect of dexmedetomidine on postoperative delirium in patients undergoing awake craniotomies: study protocol of a randomized controlled trial

**DOI:** 10.1186/s13063-023-07632-2

**Published:** 2023-09-25

**Authors:** Muhan Li, Minying Liu, Qianyu Cui, Min Zeng, Shu Li, Liyong Zhang, Yuming Peng

**Affiliations:** Department of Anesthesiology, Beijing Tiantan Hospital, Capital Medical University, Beijing, China

**Keywords:** Dexmedetomidine, Postoperative delirium, Awake craniotomy

## Abstract

**Introduction:**

Postoperative delirium (POD) is a common complication, and it has a high incidence in neurosurgery patients. Awake craniotomy (AC) has been widely performed in patients with glioma in eloquent and motor areas. Most of the surgical procedure is frontotemporal craniotomy, and the operation duration has been getting longer. Patients undergoing AC are high-risk populations for POD. Dexmedetomidine (Dex) administration perioperatively might help to reduce the incidence of POD. The purpose of this study is to investigate the effect of Dex on POD in patients undergoing AC.

**Methods:**

The study is a prospective, single-center, double-blinded, paralleled-group, randomized controlled trial. Patients undergoing elective AC will be randomly assigned to the Dex group and the control group. Ten minutes before urethral catheterization, patients in the Dex group will be administered with a continuous infusion at a rate of 0.2 µg/kg/h until the end of dural closure. In the control group, patients will receive an identical volume of normal saline in the same setting. The primary outcome will be the cumulative incidence and severity of POD. It will be performed by using the confusion assessment method in the first 5 consecutive days after surgery. Secondary outcomes include quality of intraoperative awareness, stimulus intensity of neurological examination, pain severity, quality of recovery and sleep, and safety outcomes.

**Discussion:**

This study is to investigate whether the application of Dex could prevent POD in patients after undergoing AC and will provide strong evidence-based clinical practice on the impact of intraoperative interventions on preventing POD in AC patients.

**Trial registration:**

ClinicalTrials.gov, NCT05195034. Registered on January 18, 2022.

**Supplementary Information:**

The online version contains supplementary material available at 10.1186/s13063-023-07632-2.

## Background

Delirium is an abrupt change in the brain characterized by acute onset of a change in mental status, inattention, disturbance in thinking, and an altered level of consciousness. Postoperative delirium (POD) is a significant complication following surgery [1]. POD may result in longer hospitalization, long-term postoperative cognitive dysfunction, and increasing mortality [2–4].

As high-risk patients, the incidence of POD is as high as 10 to 40% in neurosurgery patients [5]. In particular, a prospective cohort study reported that the incidence of POD was about 30% in ICU patients after elective intracranial surgery [6]. Moreover, in a recent randomized controlled trial (RCT), POD was recurred in 25% of patients undergoing cranial surgery [7]. However, these studies involved a wide variety of types of neurosurgery and were not limited to brain tumor patients. Chen H et al. conducted a cross-sectional survey and found that the incidence of POD was about 15% after brain tumor resection [8]. Patients with brain tumors are a high-risk population for POD. Pathological diagnosis, tumor site, and duration of anesthesia/operation were risk factors for POD in a study of 916 patients undergoing brain tumor resection [8]. Wang CM et al. showed that frontal approach craniotomy was the independent risk factor for POD in patients admitted to the ICU after neurosurgery [6].

Awake craniotomy (AC) has been considered as routine care for primary gliomas, located within or close to the functional areas (i.e., sensorimotor or language areas) [9]. Nowadays modern AC techniques combined with intraoperative neurophysiologic monitoring and cortical mapping allow the surgeon to identify exactly the eloquent areas for individual patients, aiming for the maximal removal of lesions without any significant neurological deficit. Therefore, AC could provide a wider extent of mass without postoperative neurological impairment and improve the survival rates of patients [10]. However, tumor resection in eloquent areas increases the risk of postoperative cognitive dysfunction [11]. Gliomas, in particular, damage the surrounding brain tissue because of their malignant behaviors. Excessive craniotomy and longer operation duration can cause an aberrant stress response in patients and damage the blood–brain barrier [12], distributing inflammatory factors into the brain, which triggers POD occurrence [13]. As mentioned above, tumors in the supratentorial area and frontal approach craniotomy were the influencing factors for POD, which was the common surgical site involved in AC. Therefore, AC patients are also potential candidates for POD.

Dexmedetomidine (Dex) is a selective alpha 2 adrenergic agonist with sympathetic effects that reduce heart rate, thereby improving cardiac oxygen consumption [14]. At the same time, it has an analgesic effect and reduces the patient's demand for opioids and other sedative drugs [15]. Some meta-analyses indicated that perioperative Dex administration significantly reduced the risk of POD in ICU or surgical patients [16, 17]. However, these study populations did not involve neurosurgical patients. Chen PH et al*. *[7] revealed that the application of Dex (infusion rate of 0.5 µg/kg/h) did not reduce the incidence of delirium in patients undergoing cranial surgery. This study included various types of neurosurgeries, not only focusing on tumor resection. In addition, compared to propofol, Dex administered with continuous infusion at a rate of 0.1–0.4 µg/kg/h intraoperatively has been shown to be safe for AC patients and had no influence on the quality of intraoperative brain mapping and efficacy of sedation [18]. However, the effect of intraoperative Dex infusion on POD in AC patients is still unclear.

Therefore, we propose the hypothesis that intraoperative administration of Dex reduces the incidence of POD in patients undergoing AC. We will conduct a RCT to test the hypotheses. In this RCT, the primary outcome will be the incidence of POD in the first 5 consecutive days after surgery. The secondary outcomes will include quality of intraoperative awareness, stimulus intensity of neurological examination, pain severity, quality of recovery and sleep, and safety outcomes.

## Methods

### Study design {8, 9}

**Fig. 1:**
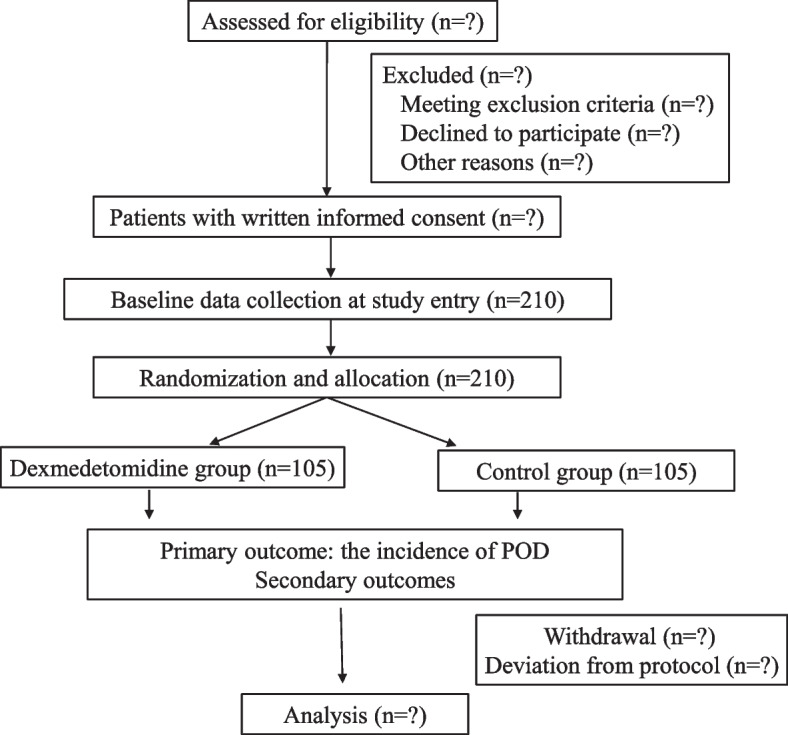
Flowchart of enrollment, interventions, and assessments. POD, postoperative delirium

This single-center, double-blinded, randomized controlled trial with two parallel arms is designed to explore whether Dex prevents POD in patients undergoing awake craniotomy. The study will be conducted at Beijing Tiantan Hospital, Capital Medical University, which was approved by the Medical Ethics Committee of Beijing Tiantan Hospital (No. KY2022-002–02) and was registered on ClinicalTrials.gov on 18 January 2022 (NCT05195034). A brief flow diagram of the entire study is shown in Fig. 1.

### Study population {10}

Patients older than 18 years and scheduled for elective awake craniotomy will be screened for eligibility 1 day before surgery.

Exclusion criteria include preoperative moderate and severe cognitive impairment (Montreal Cognitive Assessment, MoCA < 18); preoperative psychotropic medication history within 1 year; BMI ≤ 18 or ≥ 30 kg/m^2^; pregnant or lactating women; history of traumatic brain injury or neurosurgery; severe bradycardia (heart rate less than 40 bpm); sick sinus syndrome or second- to third-degree atrioventricular block; and severe hepatic or renal dysfunction.

### Patient and public involvement

Patients or the public are not involved in the design or conducting of our research. At the completion of the study, a manuscript will be prepared to present the trial results. The results of the final study will be disseminated to all study participants through the methods indicated at the time of enrollment.

### Randomization and blinding {16, 17}

Random numbers will be conducted based on a computer-generated table by an independent research assistant with a block size of 6. The results of randomization will be sealed in sequentially numbered envelopes and stored at the site of study until the end of the study. Access to the allocation sequence is limited to the study investigators who do not participate in clinical anesthesia management and outcome assessment. The researcher will open the envelopes and clarify the group assignment on the day of surgery. The researchers who are unaware of the allocation sequence will screen and enroll participants. Patients who will meet the inclusion and exclusion criteria and give written informed consents will be randomly divided into two groups with a 1: 1 ratio on the day of surgery. Allocation will be concealed until the database is locked.

The enrolled patients, anesthesiologists, and outcome assessors will all be blinded to the allocation until the study analysis is completed. We do not anticipate any requirement for unblinding, but if required, the principal investigator or the data manager will have access to group allocations and any unblinding will be reported.

### Intervention and grouping {11a-c}

Ten minutes before urethral catheterization, patients in the Dex group will be administered with continuous infusion at a rate of 0.2 µg/kg/h until the end of dural closure. In the control group, patients will receive an identical volume of normal saline in the same setting. If patients have severe bradycardia or hypotension that cannot be treated with medication during the study period, the trial drug will be temporarily or permanently stopped. If patients fail to be awaken, the trial drug will be reduced or discontinued. Anesthesiologists will be responsible for using trial drugs and making final decisions. The cause of the above situations as well as the duration and dose of trial drug application will be recorded. At the same time, these patients will be entered into the per-protocol analysis set.

The intervention of this study is conducted intraoperatively, and the attending anesthesiologist on the day of surgery carries out the intervention according to the standard operating procedure (SOP) to improve adherence to intervention protocols.

### Anesthesia management {11d}

For each patient, standard monitoring will be established, including body temperature, bispectral index (BIS), electrocardiograph (ECG), end-tidal carbon dioxide PaO2 (ETCO_2_), non-invasive blood pressure (NIBP), pulse oxygen saturation (SpO_2_), continuous arterial pressure and urine output. Pre-anesthesia medication, including penehyclidine hydrochloride 0.5 mg, ondansetron hydrochloride 4–8 mg, and sufentanil 5–10 µg, will be given intravenously. Urethral catheterization will be then performed in awake patients.

**Fig. 2:**
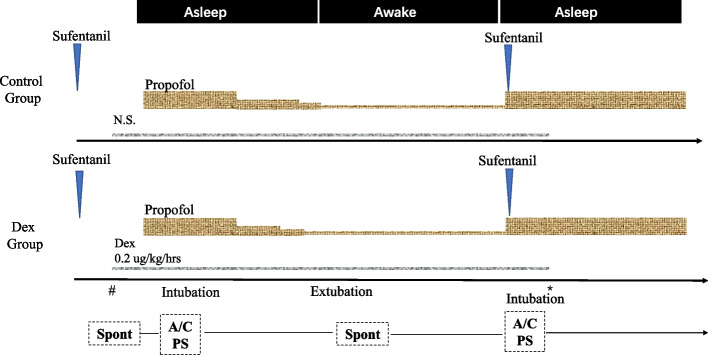
Anesthesia management of asleep-awake-asleep (SAS) technique. N.S., normal saline; Dex, dexmedetomidine; #, 10 min before urethral catheterization; *, the end of dural closure

In the first asleep stage, anesthesia will be induced with propofol (target-controlled infusion, TCI) 3–5 µg/ml. When the BIS value has decreased to 40–50, the laryngeal mask will be intubated. Then mechanical ventilation will be established with a tidal volume of 6 to 8 mL/kg, a respiratory rate of 12 to 15/min, and a 1:1 air-oxygen mixture. The ETCO_2_ will be maintained between 35 and 45 mmHg. The cranial nerve block for six nerves and the regional scalp block along with incision line infiltration will be performed with 0.5% ropivacaine before surgery. For anesthesia maintenance, the TCI concentration of propofol will be maintained BIS value between 50 and 60. In the awake stage, the TCI concentration of propofol will be reduced to about 0.5ug/ml. After the patient's spontaneous respiration recovered and the BIS value recovered to about 70 to 80, the laryngeal mask will be extubated. Then patients will be awakened and evaluated for their motor and language function. The TCI concentration of propofol will be adjusted according to the BIS value during the intraoperative awake period. In the second asleep stage, after tumor resection, patients will be sedated again with propofol (TCI 3–5 µg/ml) and sufentanil (0.05–0.1 μg/kg) intravenously (Fig. 2). No inhalational agent and remifentanil will be used.

Heart rate and mean arterial pressure will be maintained within ± 20% of baseline. Inhaling anesthetics, muscle relaxants, and remifentanil will not be used in this study. Postoperative patient-controlled intravenous analgesia (PCIA) regimen will be established with sufentanil (1 µg/ml) combined with ondansetron (0.16 mg/ml), programmed to deliver a 0.5-mL bolus with a lockout interval of 15 min and a basal infusion of 2 mL/h. The patient will be delivered to the post-anesthesia care unit after the surgery.

### Data collection {18}

Preoperative data including demographics, medical history, medication history, supplementary examination, and preoperative assessment will be collected by an independent research assistant. Intraoperative data including doses of anesthetics, analgesics, and other drugs used during anesthesia, the type of surgery, duration of anesthesia and surgery, fluid balance, and transfusion of blood products will be recorded by the anesthesiologists. Physiological variables will be recorded every 15 min manually at the critical time points and every 10 s electronically during the operation. The outcome assessment will be performed by the trained research assessors. Personal information of all participants will be confidentially kept.

### Primary and secondary outcomes {12}

The primary outcome is the incidence of POD during the first 5 days after surgery. Delirium will be assessed two times per day (8:00–9:00 and 16:00–17:00) during postoperative days 1–5. Almost all AC patients will be delivered to the general ward after surgery. The patients will be assessed by the Richmond Agitation Sedation Scale (RASS) in the first step [19]. If the score is less than − 3, the remaining assessment will be discontinued, and the patient will be recorded as comatose. When the RASS score is equal to or higher than − 3, then delirium will be evaluated by the 3-min diagnostic interview for CAM (3D-CAM) in the second step. 3D-CAM includes 20 items that best operationalize the 4 CAM diagnostic features and provide a brief, sensitive, and specific delirium assessment tool in general units [20]. For patients delivered to ICU, POD will be judged by RASS and the confusion assessment method for ICU (CAM-ICU) [21]. At the same time, the severity of delirium has been already assessed by using the Delirium Rating Scale-Revised-98 (DRS-R-98) in our study. The scale includes 13 items with each score ranging from 0 (no symptoms) to 3 (most severe symptoms). Each of the items is related to different symptoms. The total score ranges from 0 to 39, and the higher the score, the more serious the delirium symptoms are. The assessors will be trained by psychiatrists before the study initiation.

The secondary outcomes include other efficacy and safety outcomes.

1. Quality of intraoperative awareness: RASS, nociceptive, and emotional domain of recovery included in the Post-operative Quality Recovery Scale (PQRS) will be administered to assess intraoperative awareness at 5 min, 10 min, 30 min, 1 h, 2 h, 3 h, and 4 h after awakening the patients up [19, 22].
2. Neuromonitoring: the minimum and maximum stimulus intensity of intraoperative neuromonitoring and electrocortical stimulation in motor, somatosensory, and language will be recorded after induction and at awake time. The ability to complete the brain mapping will also be recorded.
3. The duration of restoring consciousness is defined as the time from decreasing the concentration of propofol to consciousness recovery and will be recorded.
4. Quality of recovery: it will be assessed by a 15-item quality of recovery questionnaire (QoR-15) on postoperative day 1 [23]. QoR-15 contains 15 items with scores ranging from 0 (extremely poor recovery) to 150 (all, excellent recovery).
5. Degree of tumor resection: it will be divided into three grades, in which grade 1 indicates macroscopically complete removal. The higher the grade, the higher the rate of residual tumor is.
6. Pain severity: numerical rating scale (NRS) will be used to evaluate pain severity at rest and on movement from the period of emergence to the first 3 days after surgery [24]. The score of NRS ranges from 0 (no pain) to 10 (the worst pain).
7. Quality of sleep: this outcome will be assessed by the Richards Campbell sleep questionnaire (RCSQ) from the first to the third day after surgery [25]. The total score of RCSQ is divided into five items, in which each of them ranges from 0 (extremely poor sleep quality) to 100 (excellent sleep quality).
8. Intraoperative adverse events: the incidence of seizure and AC failure, hypotension (systolic blood pressure < 95 mmHg or a decrease of more than 30% from baseline), hypertension (systolic blood pressure > 180 mmHg or an increase of more than 30%), bradycardia (defined as heart rate < 40 bpm), tachycardia (heart rate ≥ 100 bpm) or hypoxemia (pulse oxygen saturation < 90%). In addition, the incidence of agitation and emergence of delirium during the awake phase will be recorded in our study.
9. The incidence of non-delirium complications within 5 days after surgery, including myocardial infarction, pulmonary infection or embolism, intracranial hematoma or severe edema, and surgical site infection.
10. Length of stay in ICU and hospital.
11. All-cause 30-day mortality.

### Data management and monitoring {19}

**Fig. 3:**
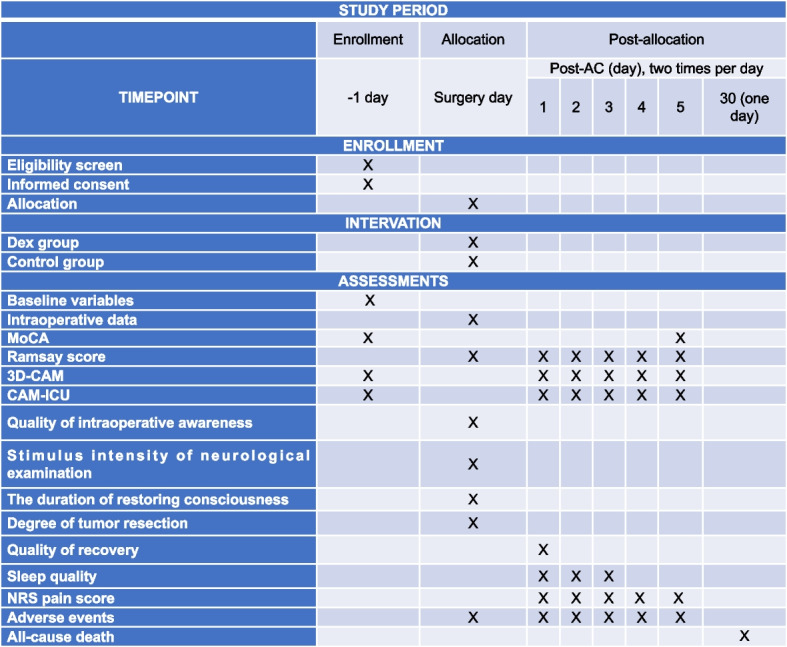
Data collection at each time point. Post-AC, post-awake craniotomy; Dex, dexmedetomidine; MoCA, Montreal Cognitive Assessment; 3D-CAM, 3-min diagnostic interview for CAM; CAM-ICU, confusion assessment method for ICU

The data collection of events at each time point is provided in Fig. 3. The whole research team members will be required to attend the professional training before recruitment and strictly adhere to the study protocol. All the raw data will be recorded in the case report forms. Data will be entered doubly performed by two persons and monitored securely in an electronic database with password protection at the medical center. The database will be locked after all data have been cleaned. All the original files will be maintained in storage for 5 years after completion of the study.

### Reporting of adverse events {22}

Adverse events (AEs) are any undesirable experiences the participants endure during the study period, whether or not considered intervention-related. In this study, the AEs of Dex will be closely monitored from the start of infusion to the 5th day after the surgery. All details of the AEs, such as the type, the diagnosis time, the duration, management, and the consequences, will be recorded by the investigators. All AEs will be followed up from the beginning to the resolution. The principal investigator will be informed of any serious AEs and judge the severity and causality of these events. All AEs associated with the study will be recorded and reported to the ethics committee as part of the annual report.

### Sample size estimation {14} and statistical analysis {20}

According to our previous study, the incidence of delirium after frontotemporal tumor resections is about 35% [26]. A meta-analysis indicated that Dex relatively alleviated the incidence of delirium by approximately 54% [27]. Therefore, we hypothesize that the incidence of POD is 35% and could be reduced by 50% after administration of Dex compared with the placebo. With a significance and power set at 0.05 (two-sided) and 80%, respectively, the sample size required to detect difference is 97 patients in each group. Considering a 5% loss in follow-up, 210 patients will be needed to be enrolled.

The analyses will be performed on an intention-to-treat basis according to group allocation and will be done by using SPSS software (V.22.0). The continuous variables will be presented with a mean (SD) or median (IQR) and analyzed using *t* test or the Mann–Whitney *U* test. Categorical data will be described with counts (percentage) and analyzed by chi-square tests or Fisher’s exact test.

The primary outcome, compared with the difference in cumulative incidence of POD between the two groups, will be analyzed by the chi-square test and also be analyzed in the subgroups, including age (more than 65 years or not), gender, duration of surgery, and tumor size [6]. And the primary outcome will be analyzed per protocol.

Baseline characteristics are presented as numbers and percentages in Dex and placebo patients, along with absolute standardized differences (ASD) which are defined as absolute differences in means, mean ranks, or proportions divided by the pooled standard deviation. Variables with ASD > 0.254 = ($1.96* \sqrt{\frac{1}{105}+\frac{1}{105}}$), where 105 is the number of patients in each group, will be considered imbalanced and adjusted for in the primary outcome analysis. We plan to use multivariable methods to adjust for unbalanced baseline factors.

The changes in the quality of intraoperative awareness (RASS scores), NRS scores, and the cumulative consumption of opioids will be compared by using repeated measurements. Other secondary outcomes such as stimulus intensity, QoR-15, quality of sleep, and intraoperative data will be analyzed by *t* test or Mann–Whitney *U* test. Besides, missing data will be imputed by using the worst-case imputation scenarios.

### Protocol amendment {25}

The principal investigator will be responsible for amending the protocol and making a final decision. If there is any important protocol modification (e.g., changes to eligibility criteria, outcomes, analyses), the principal investigator will communicate and gain approval from the local Medical Ethics Committee prior to implementation.

### Frequency and plans for auditing trial conduct {21a, 23}

There is no Data Monitoring Committee (DMC) in this study because it is a single-center RCT with a relatively small sample size and low-risk intervention. The Trial Steering Group is responsible for running the trial day-to-day and providing organizational support, consisting of a principal investigator, research assistant, recruitment, and assignment personnel. They will meet to review every 6 months and the ethics committee will perform an annual follow-up review.

### Interim analyses and stopping guidelines {21b}

No interim analyses are planned. The study will be stopped when the enrollment is completed if no serious adverse events (SAEs) occur during the study. If there are SAEs, the study will be stopped. SAEs include death, disability, and severe allergic reactions that significantly impact the patient’s health.

## Discussion

This double-blinded, paralleled-group, randomized controlled trial is to investigate whether the application of Dex could prevent POD in patients after undergoing AC.

AC, as the optimal choice for brain tumor resection of functional area, requires special anesthesia techniques. There are two dominant anesthetic approaches for AC: monitored anesthesia care (MAC) and asleep-awake-asleep (SAS) [28, 29]. The MAC technique implies low doses of sedative drugs and spontaneous ventilation, which is intended to restrict the sedative load to avoid a sharp asleep-to-awake transition. In contrast, the SAS technique is commonly used in higher doses of anesthetics for achieving deep sedation as well as mechanical ventilation. The core of this approach is to provide better comfort for the patient before awake and be advantageous for controlling brain swelling via hyperventilation. Despite the significant differences in these two methods, in some retrospective analyses conducted in multicenter or a single-center, both of them can lead to success for intraoperative mapping and be safe for AC patients [30, 31]. In another recent retrospective analysis comparing the effectiveness of these two techniques, differences were more significant [32]. Higher incidence of seizures and agitation, more complaints of pain, and higher levels of PaCO_2_ were found in the MAC group than that in the SAS group. However, blood pressure was higher, and more antihypertensive treatment was required in the SAS group than that in the MAC group during the awake stage. Although these two approaches provide similar effectiveness in AC, the MAC technique needs to be performed with better cooperation among anesthesiologists, surgeons, and neurophysiologists [33]. Therefore, in our study, the SAS technique is the optimal choice, which is preferred in our institution and more applicable for AC patients.

The successful implementation of AC, defined as achieving complete awake monitoring of the brain function, requires a high degree of cooperation of patients [34]. In this context, patients need to recover consciousness without hypoactive or hyperactive emergence delirium and feel comfortable in the awake phase. Intraoperative adverse events (such as seizures, respiratory depression, and hypertension) do not happen or can be solved by simple intervention. Therefore, in our study, the RASS scale will be used to evaluate patients’ agitation and sedation [19]. At the same time, nociceptive and emotional domains of recovery included in the PQRS will assess the severity degree of pain, nausea, anxiety, and depression during the awake phase [22]. The above indicators represent the quality of intraoperative awareness. The duration of restoring consciousness and the influence on neurological examination are important indicators to study whether dexmetomidine interferes with the successful implementation of AC as well. Seizure is a serious complication encountered due to electrical cortical stimulation during brain mapping. According to a multicenter retrospective study, the frequency of seizures during AC varied widely between 2.9 and 54% [30]. Seizures can be commonly stopped by brain tissue irrigation with ice-cold saline by the surgeon. If it does not work, low doses of intravenous propofol can be recommended [28]. Airway obstruction is another serious complication, but can be easily solved by jaw thrust or supplemental oxygen. Meanwhile, a full range of airway equipment should be immediately available [28]. These adverse events and their interventions will be recorded in our study.

In order to maximize resection, it is inevitable to make larger wound surfaces and lengthen operational time, which greatly increases the risk of POD. In this study, we will evaluate POD two times a day within the postoperative 5 days by using 3D-CAM or CAM-ICU, which is highly sensitive and specific to delirium. It is necessary to explore perioperative pharmacological intervention to prevent POD. Nowadays, the efficacy and safety of Dex administered in AC have already been proven in some studies. However, the correlation between Dex and POD in AC patients has not been clear. As far as we know, the results of this RCT will provide strong evidence-based clinical practice on the impact of intraoperative interventions on preventing POD in AC patients.

## Trial status

At the time of manuscript submission, the study is in the phase of recruiting. The anticipated start date for the enrollment is March 31, 2022. It is estimated that over 50 awake craniotomies will be carried out in Beijing Tiantan Hospital every year. We expect to complete the study by December 2025.

### Supplementary Information

**Additional file 1.** SPIRIT checklist.
